# Combined isobutyryl‐CoA and multiple acyl‐CoA dehydrogenase deficiency in a boy with altered riboflavin homeostasis

**DOI:** 10.1002/jmd2.12292

**Published:** 2022-05-07

**Authors:** Albina Tummolo, Piero Leone, Maria Tolomeo, Rita Solito, Matteo Mattiuzzo, Francesca Romana Lepri, Tania Lorè, Roberta Cardinali, Donatella De Giovanni, Simonetta Simonetti, Maria Barile

**Affiliations:** ^1^ Metabolic Diseases and Clinical Genetics Unit Children's Hospital “Giovanni XXIII” Bari Italy; ^2^ Department of Biosciences, Biotechnology and Biopharmaceutics University of Bari “A. Moro” Bari Italy; ^3^ Laboratory of Medical Genetics Translational Cytogenomics Research Unit, Bambino Gesù Children Hospital Rome Italy; ^4^ Regional Centre for Neonatal Screening Children's Hospital “Giovanni XXIII” Bari Italy

**Keywords:** *ACAD8*, *ETFDH*, IBDD, MADD, RFVT2, riboflavin

## Abstract

In this report, we describe the case of an 11‐year‐old boy, who came to our attention for myalgia and muscle weakness, associated with inappetence and vomiting. Hypertransaminasemia was also noted, with ultrasound evidence of hepatomegaly. Biochemical investigations revealed acylcarnitine and organic acid profiles resembling those seen in MADD, that is, multiple acyl‐CoA dehydrogenase deficiencies (OMIM #231680) a rare inherited disorder of fatty acids, amino acids, and choline metabolism. The patient carried a single pathogenetic variant in the *ETFDH* gene (c.524G>A, p.Arg175His) and no pathogenetic variant in the riboflavin (Rf) homeostasis related genes (*SLC52A1*, *SLC52A2*, *SLC52A3*, *SLC25A32*, *FLAD1*). Instead, compound heterozygosity was found in the *ACAD8* gene (c.512C>G, p.Ser171Cys; c.822C>A, p.Asn274Lys), coding for isobutyryl‐CoA dehydrogenase (IBD), whose pathogenic variants are associated to IBD deficiency (OMIM #611283), a rare autosomal recessive disorder of valine catabolism. The c.822C>A was never previously described in a patient. Subsequent further analyses of Rf homeostasis showed reduced levels of flavins in plasma and altered FAD‐dependent enzymatic activities in erythrocytes, as well as a significant reduction in the level of the plasma membrane Rf transporter 2 in erythrocytes. The observed Rf/flavin scarcity in this patient, possibly associated with a decreased ETF:QO efficiency might be responsible for the observed MADD‐like phenotype. The patient's clinical picture improved after supplementation of Rf, l‐carnitine, Coenzyme Q10, and also 3OH‐butyrate. This report demonstrates that, even in the absence of genetic defects in genes involved in Rf homeostasis, further targeted molecular analysis may reveal secondary and possibly treatable biochemical alterations in this pattern.

**SYNOPSIS**

Secondary and possibly treatable biochemical alterations linked to Rf homeostasis can be revealed by targeted molecular analysis, even in the absence of a genetic defect in the involved genes.

## 1 INTRODUCTION

To perform the terminal metabolism of fatty acids and amino acids, several flavin‐dependent oxidoreductases must be functionally active in the mitochondria of tissues with high energy demand, such as muscle. A prominent role in these pathways is played by different acyl‐CoA dehydrogenases (ACADs) involved in the beta‐oxidation of fatty acids: that is, short‐chain (SCAD), medium‐chain (MCAD), long‐chain (LCAD), very‐long‐chain (VLCAD) acyl‐CoA dehydrogenases, and novel members of the same family, that is, ACAD10 and 11. Besides involvement in beta‐oxidation, an additional chaperoning role was recently proposed for ACAD9.^1^ Four distinct ACADs allow for specific amino acid catabolism: isobutyryl‐CoA (IBD), isovaleryl‐CoA (IVD), glutaryl‐CoA, and short/branched‐chain acyl‐CoA dehydrogenases. The majority of mitochondrial ACADs have common features: they bind FAD very tightly, but not covalently, and are tetrameric molecules composed of protomers of about 40 kDa. Exceptionally, VLCAD and ACAD9 are homodimers of about 70 kDa,^2^ ACAD10 and 11 protomers perform molecular weights of 90 kDa and 118 kDa, respectively (see Mosegaard and colleagues^1^, ^3^, ^4^, ^5^ for extensive reviews). Electrons deriving from all these flavin‐dependent ACADs are funneled to the respiratory chain through the system of two inner membrane located flavoenzymes, that is, the peripheral electron transfer flavoprotein (ETF), that physically enters in contact with ACADs^5^, ^6^ and its dehydrogenase ETFDH or ETF:QO (ETF dehydrogenase or oxidoreductase; EC 1.5.5.1) embedded in the membrane and driving electrons to ubiquinone.

Homozygous or compound heterozygous mutations in *ETF* and *ETFDH* are responsible for a rare inborn error of metabolism named multiple acyl‐CoA dehydrogenase deficiency (MADD) or glutaric aciduria of type II (OMIM #231680) characterized by a disorder of fatty acids and certain amino acids metabolism, abnormal acylcarnitine plasmatic profile, and secondary carnitine deficiency. Its clinical presentation is heterogeneous, ranging from a severe, neonatal form, characterized by hypoketotic hypoglycemia, metabolic acidosis, cardiomyopathy, and hepatomegaly with or without congenital anomalies^7^ to a mild or late‐onset form, essentially characterized by proximal myopathy. Some of these patients respond to pharmacological treatment with riboflavin (Rf) and are referred to as Rf‐responsive MADD patients.

Recently, mutations in genes involved in Rf transport and metabolism, that is, Rf transporter 1 gene (*SLC52A1*),^1^, ^8^, ^9^ FAD synthase gene (*FLAD1*),^10^ and mitochondrial FAD transporter gene (*SLC25A32*)^11^, ^12^ have been shown to cause MADD(‐like) clinical and biochemical phenotypes. Mutations in Rf transporter 3 gene (*SLC52A3*)^13^ and Rf transporter 2 gene (*SLC52A2*),^14^ which have been shown to cause Brown–Vialetto–Van Laere or Fazio Londe syndromes, might lead to a MADD(‐like) biochemical profile (see Tolomeo and colleagues^15^, ^16^, ^17^). This is essential because FAD, synthesized from Rf, is the main cofactor for the functioning of ETF, ETF:QO and all ACADs.

Here, we describe a case of an 11‐year‐old boy, who revealed clinical symptoms, acylcarnitine, and organic aciduria profiles resembling late‐onset MADD, harboring a pathological variant in *ETFDH* gene and no pathological variants in flavin homeostasis related genes, despite altered plasma levels of flavin cofactors and FAD‐dependent enzymatic activities. We, indeed, found compound heterozygous mutations in the *ACAD8* gene, which codes isobutyryl‐CoA dehydrogenase (IBD; EC 1.3.8._), one member of the ACAD family, which catalyzes the conversion of isobutyryl‐CoA to methacrylyl‐CoA in the third step of valine catabolism^18^ (Figure S1).

## 2 MATERIALS AND METHODS

### 2.1 Ethics statement

Informed consent for participation in this study was obtained from the parents of investigated subject and all procedures were performed in agreement with the Declaration of Helsinki. Ethics committee approval was not necessary for the study.

### 2.2 Biochemical analysis

Plasma acylcarnitine and amino acid profiles were performed by liquid chromatography (LC)‐MS/MS.^19^ Urine organic acids and acylglycines were analyzed by gas chromatography/mass spectrometry (GC/MS).^20^

### 2.3 Genetic analysis

Next‐generation sequencing was conducted on DNA extracted from blood leukocytes using standard procedures. Twist Human Core Exome Kit (Twist Bioscience) according to the manufacturer's protocol and sequenced on the Illumina NovaSeq 6000 platform. The BaseSpace pipeline (Illumina) and the TGex software (LifeMap Sciences) were used for variant calling and annotating variants, respectively. Sequencing data were aligned to the hg19 human reference genome. The functional impact of variants was analyzed by Combined Annotation Dependent Depletion V.1.3, Sorting Intolerant from Tolerant (SIFT), and Polymorphism Phenotyping v2 (PolyPhen‐2). Rare variants with minor allele frequency <0.1% were filtered based on the Genome Aggregation Database. Based on the guidelines of the American College of Medical Genetics and Genomics (ACMG, S1^21^), a minimum depth coverage of 30X was considered suitable for analysis. Variants were examined for coverage and *Q* score (minimum threshold of 30) and visualized by the Integrative Genome Viewer (IGV). Library preparation and exome capture were performed using the Twist Human Core Exome Kit (Twist Bioscience) according to the manufacturer's protocol and sequenced on the Illumina NovaSeq 6000 platform. The BaseSpace pipeline (Illumina) and the TGex software (LifeMap Sciences) were used for variant calling and annotating variants, respectively. Sequencing data were aligned to the hg19 human reference genome. Mutational analysis was focus to a in silico gene panel that includes the coding regions and splice junctions of genes associated to biochemical phenotype: *ETFA* (NM_000126), *ETFB* (NM_001985), *ETFDH* (NM_004453), *ACAD8* (NM_014384), *ACADS* (NM_000017), *SLC52A1* (NM_005984), *SLC52A2* (NM_001253816), *SLC52A3* (NM_033409), *FLAD1* (NM_025207), SLC25A32 (NM_030780). Based on the guidelines of the American College of Medical Genetics and Genomics (ACMG, S1^21^), a minimum depth coverage of 30X was considered suitable for analysis. Variants were examined for coverage and *Q* score (minimum threshold of 30) and visualized by the IGV.

### 2.4 Blood and plasma samples

Blood samples were collected into evacuated tubes containing K3EDTA. The plasma and packed red blood cells were separated essentially as in the study by Habif et al.^22^ One millimolar phenylmethylsulphonyl fluoride (PMSF; as proteases inhibitor) was added to plasma, which was immediately used or stored at −80°C until flavin content analysis was performed.

Aliquots (100–200 μl) of packed red blood cells were washed three times with one volume of 9.0 g/L NaCl, centrifuged at 3500 × *g* for 10 min and the supernatant was discharged. The washed red blood cells were then osmotically lysed by adding four volumes of lysis buffer (5 mM Tris–HCl pH 7.5, 1 mM PMSF, and 22 μM Leupeptin), on ice for 5 min, and then centrifuged at 5000 × *g* for 10 min. The supernatant (hemolysate) was used for enzymatic assays and hemoglobin content measurements, essentially as in the study by Bhaskaram et al.^23^; the enriched‐membrane crude pellet was frozen at −80°C until Western blotting analyses were performed.

### 2.5 Flavin content analysis

For flavin content analysis, 120 μl of either fresh or defrozen plasma were added with 2 mM NaF (as phosphatase inhibitor) and then deproteinized by treatment with 10% perchloric acid as described in the studies by Giancaspero and colleagues.^24^, ^25^ Rf, FMN, and FAD were measured in neutralized perchloric acid extracts of plasma samples by HPLC as described in the study by Leone et al.^26^

### 2.6 Erythrocytes glutathione reductase activation coefficient

Glutathione reductase (GR; EC. 1.6.4.2) is an important flavoenzyme, which in erythrocytes catalyzes the reduction of oxidized glutathione (GSSG) in the presence of NADPH. Erythrocytes GR activation coefficient (EGRAC) represents the degree of stimulation of the endogenous reaction rate resulting from the *in vitro* addition of FAD to hemolysates and it is a good indicator of the nutritional Rf status. Our measurements were carried out essentially as in the studies by Habif and colleagues,^22^, ^27^ with some modifications. Briefly, 5–10 μl hemolysates were added with or without the cofactor FAD (20 μM) at 37°C. Thirty minutes later, the hemolysates were transferred in 1 ml of 0.1 M potassium phosphate buffer (pH 7.0), EDTA 1 mM, KCl 0.2 M, at 25°C, in the presence of 0.1 mM NADPH; the reaction started with the addition of GSSG (1 mM) and its rate was measured by following the change in optical density at 340 nm due to NADPH oxidation by using of an Ultrospec 3100 Spectrophotometer (Amersham Biosciences).

### 2.7 Sodium dodecyl sulfate–polyacrylamide gel electrophoresis and Western blotting

Enriched‐membrane erythrocyte fractions (deriving from 100–200 μl cells) were dissolved in sodium dodecyl sulfate–polyacrylamide gel electrophoresis (SDS‐PAGE) loading buffer and separated on 10% T polyacrylamide gels, according to Laemmli.^28^ Separated proteins were electrotransferred onto a PVDF membrane using a transblot semidry electrophoretic transfer cell (Sigma‐Aldrich). The immobilized proteins were incubated overnight with polyclonal antibodies against hRFVT1, 2, and 3 (1:2000 dilution; Thermo Fisher Scientific). A monoclonal anti‐β‐actin antibody (1:10000 dilution; Sigma‐Aldrich) was used to reveal and quantify protein markers. The bound antibodies were visualized with the aid of secondary anti‐rabbit IgG antibodies conjugated with peroxidase (1:2500 dilution). Densitometry was performed using the Image Lab software (Bio‐Rad) of the ChemiDoc (Bio‐Rad) imaging system.

### 2.8 Statistical analysis

All the results were reported as mean ± SEM. Student's *t*‐test was used for the comparison of means between patients and controls.

## 3 RESULTS

### 3.1 Case presentation

The patient was an 11‐year‐old boy who came for the first time to our attention, for muscle pain and weakness, associated with inappetence and vomiting. Hypertransaminasemia (AST 144 IU/L, ALT 267 IU/L), with echographic evidence of hepatomegaly, was the reason for admission to our division.

The second son of two siblings, his parents were healthy and unrelated. He was born at full term from spontaneous delivery, bodyweight 3290 g, and good extrauterine adaptation. He was breastfed until 3 years of age, and psychomotor development and stature‐weight trend were reported to be normal. Episodes of asthma were reported in the first years of life. The patient was first hospitalized at the age of 1 year for hypertransaminasemia and anemia, no further investigations were performed at that time. Two other hospitalizations, at age 4 and 5 years were due to the onset of wheezing and/or asthma exacerbation.

Since the age of 6 years, he began to manifest episodes of muscle cramps in apyrexia (normal EEG), easy fatigue, and muscle pains after light exercise. These episodes were sometimes accompanied by vomiting. In the last episode, he came to our attention; a decrease in free carnitine and a significant increase in acylcarnitines from short to very long‐chain was found, together with glutaric and 2‐ketoglutaric aciduria, but also ethylmalonic and dicarboxylic aciduria (Table 1, first decompensation on admission). At this time hypertransaminasemia (AST 90 UI/L, ALT 143 UI/L), hyperCPKemia (348 UI/L), and hyperammonemia (93 μmol/L) were also detected (Table 1, first decompensation on admission). The plasma amino acid profile revealed no significant alteration except for a marked hyperprolinemia (520.1 μmol/L, n.v. 40–332) and a slight hyperasparaginemia (232.8 μmol/L, n.v. 85–176). Neurological examination was characterized by preserved consciousness, diffuse asthenia with reduced counter‐resistance force of the lower limbs, and modest muscular hypotrophy. Heart echocardiography revealed mild concentric hypertrophy of the left ventricle. The liver appeared diffusely hyperechoic as for steatosis.

**TABLE 1:** Biochemical investigations in patient

Newborn screening: not done, because not active in 2009 in the Apulia region									
	First decompensation (on admission)	First decompensation (on discharge)	Out‐patient clinic (I)	Second decompensation (on admission)	Second decompensation (on discharge)	Out‐patient clinic (II)	Out‐patient clinic (III)	Out‐patient clinic (IV)	Out‐patient clinic (V)
	P ^1st^D_adm_	P ^1st^D_dis_	P OC (I)	P ^2nd^D_adm_	P ^2nd^D_dis_	P OC (II)	P OC (III)	P OC (IV)	P OC (V)
Therapy	None	CoQ10: 30 mg/day, l‐carnitine: 3 g/day.	CoQ10: 30 mg/day, l‐carnitine: 3 g/day.	CoQ10: 30 mg/day, l‐carnitine: 3 g/day.	Rf: 150 mg/day, CoQ10: 100 mg/day, l‐carnitine: 3 g/day.	Rf: 200 mg/day, CoQ10: 100 mg/day, l‐carnitine: 3 g/day.	Rf: 200 mg/day, CoQ10: 100 mg/day, l‐carnitine: 3 g/day.	Rf: 200 mg/day, CoQ10: 100 mg/day, l‐carnitine: 3 g/day. 3OH‐butyrate: 1200 mg/day.	Rf: 200 mg/day, CoQ10: 100 mg/day, l‐carnitine: 3 g/day. 3OH‐butyrate: 200 mg/day.
Hypolipidic hypoproteic diet	No	Partial compliance	Partial compliance	Partial compliance	Improved compliance	Good compliance	Good compliance	Good compliance	Good compliance
Routine laboratory tests – normal range in brackets									
NH_3_ μmol/L (11–32)	**93**	**34**	–	**58**	**65**	–	–	**34**	**36**
AST U/L (15–37)	**90**	**952**	–	**282**	**139**	–	–	n.v.	n.v.
ALT U/L (12–78)	**143**	**1225**	–	**279**	**408**	–	–	n.v.	n.v.
CPK U/L (31–152)	**348**	**1935**	–	**854**	n.v.	–	–	n.v.	**193**
LDH U/L (141–230)	**334**	**1713**		**730**	**819**	–	–	**238**	**236**
Plasma acylcarnitine profile (μmol/L) – normal range in brackets									
C0 (9.5–69)	6.36	53	55.39	20.37	**87**	44.06	44.28	37.13	24.85
C2 (3.5–54)	4.58	14	16.51	15.94	34	21.24	19.73	21.01	26.84
C4 (0–0.48)	**2.28**	**3.48**	**3.55**	**12.86**	**4.14**	**4.13**	**4.44**	**5.3**	**5.7**
C3DC‐C4OH (0.03–0.18)	0.02	0.05	0.04	0.05	0.05	0.04	0.05	0.06	0.08
C4/C2 (0–0.04)	**0.49**	**0.25**	**0.21**	**0.81**	**0.12**	**0.20**	**0.20**	**0.26**	**0.21**
C4/C3 (0.07–0.64)	**2.99**	**3.98**	**3.2**	**11.53**	**3.09**	**1.73**	**1.78**	**2.7**	**2.88**
C4/C8 (0.9–8.89)	n.v.	n.v.	n.v.	**17.2**	0.83	n.v.	n.v.	n.v.	n.v.
C5 (0–0.6)	n.v.	n.v.	n.v.	**2.36**	n.v.	n.v.	n.v.	n.v.	**0.98**
C5/C0 (0–0.03)	n.v.	n.v.	n.v.	**0.13**	n.v.	n.v.	n.v.	n.v.	**0.04**
C5/C2 (0–0.06)	n.v.	n.v.	n.v.	**0.15**	n.v.	n.v.	n.v.	n.v.	n.v.
C5/C3 (0.05–0.73)	n.v.	n.v.	n.v.	**2.21**	n.v.	n.v.	n.v.	n.v.	n.v.
C5OH‐C4DC (0–0.5)	n.v.	n.v.	n.v.	n.v.	n.v.	**0.58**	n.v.	n.v.	n.v.
C5DC‐C6OH (0–0.22)	n.v.	n.v.	n.v.	n.v.	n.v.	n.v.	**0.24**	n.v.	n.v.
C6 (0–0.11)	n.v.	**1.2**	**1.38**	**0.48**	**3.01**	**1.34**	**0.68**	**2.4**	**1.85**
C8 (0–0.25)	**0.26**	**2.46**	**2.13**	**0.75**	**5.01**	**3.61**	**1.81**	**3.7**	**3.84**
C10 (0–0.15)	**0.43**	**4.27**	**2.59**	**0.92**	**7.35**	**6.97**	**2.33**	**4.7**	**4.23**
C10:1 (0–0.15)	n.v.	**0.80**	**0.53**	**0.18**	**0.78**	**0.71**	**0.25**	**0.5**	**0.68**
C8/C2 (0–0.01)	**0.06**	**0.17**	**0.13**	**0.05**	**0.14**	**0.17**	**0.08**	**0.18**	**0.14**
C8/C10 (0–1.8)	n.v.	n.v.	n.v.	n.v.	n.v.	n.v.	n.v.	n.v.	n.v.
C12 (0–0.12)	n.v.	n.v.	n.v.	**0.88**	n.v.	**2.58**	**0.90**	**2.2**	**1.2**
C12:1 (0–0.08)	n.v.	n.v.	n.v.	**0.39**	n.v.	**1.10**	n.v.	**0.67**	**0.41**
C14 (0–0.27)	**0.67**	**0.65**	**0.41**	**2.13**	**1.34**	**1.0**	**0.63**	**0.97**	**0.63**
C14:1 (0–0.12)	**0.74**	**1.57**	**0.84**	**2.3**	**2.38**	**2.10**	**0.72**	**0.6**	**1.03**
C14:2 (0–0.03)	**0.13**	**0.33**	**0.15**	**0.28**	**0.41**	**0.40**	**0.12**	**0.28**	**0.19**
C16 (0.26–4.23)	n.v.	n.v.	n.v.	**5.67**	n.v.	n.v.	n.v.	n.v.	n.v.
C16:1 (0.01–0.24)	**1.12**	n.v.	**0.64**	n.v.	n.v.	**1.02**	**0.55**	**0.99**	**0.53**
C16‐OH (0–0.05)	n.v.	n.v.	n.v.	n.v.	n.v.	n.v.	n.v.	n.v.	n.v.
C16:1‐OH (0–0.08)	n.v.	n.v.	n.v.	**0.28**	n.v.	n.v.	n.v.	n.v.	n.v.
(C16 + C18:1)/C2 (0–0.37)	**0.78**	n.v.	n.v.	n.v.	n.v.	n.v.	n.v.	n.v.	n.v.
C14:1/C16 (0–0.16)	**0.45**	**0.92**	**0.69**	**0.41**	**0.60**	**0.78**	**0.32**	n.v.	n.v.
C18 (0.11–1.2)	n.v.	n.v.	n.v.	**2.05**	n.v.	n.v.	n.v.	n.v.	n.v.
C18:1 (0.23–2.4)	n.v.	n.v.	n.v.	**5.37**	n.v.	n.v.	n.v.	n.v.	n.v.
C18:2 (0–0.62)	n.v.	n.v.	n.v.	**0.98**	n.v.	n.v.	n.v.	n.v.	n.v.
C18:1‐OH (0–0.09)	n.v.	n.v.	n.v.	**0.17**	n.v.	n.v.	n.v.	n.v.	n.v.
Urine organic acids and acylglycines (GC/MS) mM/molCr – normal range in brackets									
Lactic acid (20–101)	7.6	9.8	n.v.	n.v.	9.8	n.v.	n.v.	8.5	5.5
3‐OH‐butyric acid (0–5)	0.2	0.5	0.7	2.8	0.6	0.6	0.8	1.6	9.08
Ethylmalonic acid (0.4–17)	**53.8**	**57.5**	**57.5**	**31.2**	**29.2**	n.v.	**21.2**	**30.8**	**27.9**
Fumaric acid (2–4)	**4.1**	n.v.	**5.2**	**6.4**	**4.3**	n.v.	1.5	1.6	1.5
Glutaric acid (0.5–13)	**114.9**	n.v.	n.v.	**119.8**	n.v.	n.v.	n.v.	n.v.	n.v.
Suberic acid (0–8)	**152.3**	**58.9**	**51.2**	**377.6**	**96.0**	**15.5**	**36.8**	**108.7**	**44.9**
Sebacic acid (0–8)	**34.5**	n.v.	n.v.	**56.7**	**28.7**	n.v.	n.v.	**66.2**	**12.9**
2‐OH‐glutaric acid (4–16)	**146.8**	**76.1**	**52.11**	**180.6**	**42.6**	**28.2**	**52.5**	**65.2**	**95.3**
4‐OH‐phenylacetic acid (5–70)	n.v.	n.v.	n.v.	2.7	4.2	n.v.	n.v.	n.v.	n.v.
2‐ketoglutaric acid (41–82)	**125.7**	**210.9**	**162.9**	**116.1**	**108.6**	n.v.	**94.7**	n.v.	**25.6**
Adipic acid (0–5)	**735.3**	**30.2**	**26.3**	**943.5**	**55.2**	**7.9**	**15.6**	**67.1**	**33.3**
	Trace of: acetoacetic acid, 3‐OH‐octanoic acid, 5‐OH‐octanoic acid.	Trace of: acetoacetic acid.		Trace of: 5‐OH‐hexanoic acid, 2‐OH‐sebacic acid, 3‐OH‐sebacic acid, *cis*‐4‐octanedioic acid, *cis*‐4‐decene 1,10 dioic acid.				Trace of: octanoic acid.	Trace of: acetoacetic acid.
	Trace of: butyrylglycine, isovalerylglycine, hexanoylglycine, suberylglycine.	Trace of: butyrylglycine, hexanoylglycine, suberylglycine.		Trace of: butyrylglycine, 2‐methyl‐butyrylglycine, isovalerylglycine, hexanoylglycine, suberylglycine.		Trace of: butyrylglycine, 2‐methyl‐butyrylglycine, isovalerylglycine, hexanoylglycine, suberylglycine.		Trace of: butyrylglycine, 2‐methyl‐butyrylglycine, hexanoylglycine, suberylglycine.	Trace of: butyrylglycine, 2‐methyl‐butyrylglycine hexanoylglycine, suberylglycine.

Abbreviations: –, not determined; n.v., normal value; in bold, values higher than normal ones.

Over the admission, he underwent an i.v. infusion of glucose 10% (4 mg/kg/min) and l‐carnitine (100 mg/kg), with gradual clinical recovery. He performed an in‐hospital trial with Rf (150 mg/day), with normalization of the glutaric aciduria, but persistent abnormality of the other organic acids and acylcarnitines.

The patient was discharged with a reduced intake of long‐chain lipids and controlled protein intake in his dietary regimen. l‐carnitine administration was continued per os (oral supplementation), together with Coenzyme Q10 (CoQ10). However, given the lack of biochemical response to the test (persistent dicarboxylic aciduria and elevated ethylmalonic acid) and the result of genetic analysis, Rf therapy was discontinued (Table 1, first decompensation on discharge).

At the following out‐patient clinics, partial compliance to diet was reported, and blood chemistry showed normalization of ammonium and CPK levels, although elevated levels of LDH, AST, and ALT persisted (Table 1).

About 2 months after the first admission, he presented a further metabolic decompensation characterized by vomiting, slightly numb sensory, an increase of muscle enzymes, and hyperammonemia with metabolic acidosis. The emergency protocol was performed again, with progressive clinical and biochemical improvement (Table 1, patient second decompensation).

Because of preliminary results on our Rf homeostasis studies, with evidence of significant reduction of plasma flavin levels, the patient was discharged on Rf: 150 mg/day, divided into three doses, increased to 200 mg/day 2 months later.

On follow‐up, stable clinical pattern, regular feeding, weight increase, and improved physical activity and exercise tolerance were reported. He contracted a COVID‐19 infection, with no associated symptoms.

In light of the genetic data (see below) and in the attempt to bypass the possible disturbing ketogenesis, we decided to introduce 3OH‐butyrate into therapy and maintain a higher dose of Rf. After 1 month of the above therapy, laboratory tests revealed normal muscle enzymes and ammonia levels. No significant changes in acylcarnitine profile, compared to the previous assessment, and significant dicarboxylic aciduria were detected (Table 1, out‐patient clinic [IV]).

### 3.2 Genetic analysis

To evaluate our first hypothesis of MADD we performed molecular analyses for *ETFA*, *ETFB*, and *ETFDH* genes, finding a heterozygous variant c.524G>A (p.Arg175His) in the *ETFDH* gene, which is however expected not to be responsible *per se* of the biochemical altered phenotype.^29^ This variant is listed as rs121964955 in dbSNP database (https://www.ncbi.nlm.nih.gov/snp/rs121964955). It has been classified as pathogenic in ClinVar (https://www.ncbi.nlm.nih.gov/clinvar/variation/31576/). Indeed, it is predicted to be probably damaging and tolerated *in silico* by both PolyPhen‐2 and SIFT, respectively. For *ETFDH* gene exons and flanking intronic region ±10 bp, but not deep intronic regions have been analyzed.

Rf transport and metabolism genes have been recently associated with MADD, thus, our genetic studies included sequencing of *SLC52A1*, *SLC52A2*, *SLC52A3*, *SLC25A32*, and *FLAD1* genes, in which no mutation was revealed.

DNA sequencing of *ACADS*, which codes for SCAD (EC 1.3.8.1) did not reveal any mutations.

**FIGURE 1:**
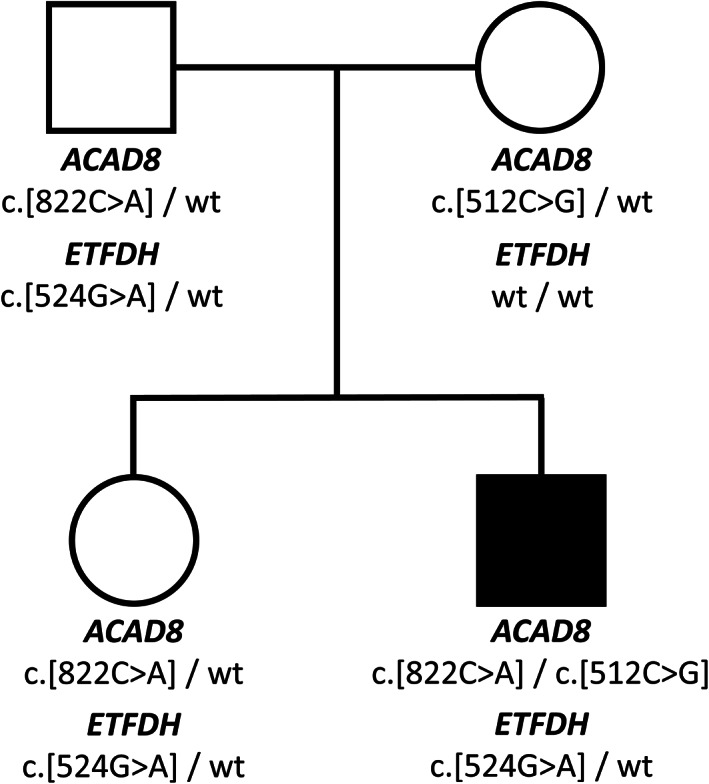
Pedigree of the family investigated in this study with *ETFDH* and *ACAD8* variants. Affected individual is indicated by closed symbol

The genetic study was expanded to other mitochondrial disorders related to the metabolism of fatty acids and amino acids. This analysis retrieved compound heterozygosity in the *ACAD8* gene, located on chromosome 11q25. These mutations, c.512C>G (p.Ser171Cys) and c.822C>A (p.Asn274Lys), affect the exons 5 and 7, respectively (Figures 1 and S2a).

As part of the family genetic counseling, we performed the molecular analysis also to the other family members: the father and sister carried the mutation in the *ETFDH* gene (c.524G>A) and one mutation in the *ACAD8* gene (c.822C>A). The mother carried the *ACAD8* mutation c.512C>G (Figure 1). Acylcarnitine profile of sister revealed higher than normal C4 levels and its related ratios (C4: 0.92 μmol/L [n.v. < 0.48], C4/C2: 0.11 [n.v. < 0.04], C4/C3: 0.71 [0.07–0.64]).

The c.512C>G (p.Ser171Cys) variant is listed as rs113488591 in dbSNP database (https://www.ncbi.nlm.nih.gov/snp/rs113488591). This variant is seen 3590 times including 40 times in the homozygous state (https://gnomad.broadinstitute.org/variant/11‐134128923‐C‐G?dataset=gnomad_r2_1). It has been reported in the literature in a patient with a compound heterozygous variant.^30^, ^31^ Still conflicting interpretations exist for the pathogenicity of this variant (https://www.ncbi.nlm.nih.gov/clinvar/variation/95589/). The Ser171 residue is highly conserved across species, it is positioned between beta‐strands 1 and 2^32^; since Ser171Cys is a nonconservative amino acid substitution (outlined in Figure S2c), it is predicted to be probably damaging and deleterious *in silico* by both PolyPhen‐2 and SIFT.

The c.822C>A (p.Asn274Lys) variant is listed as rs371156848 in dbSNP database (https://www.ncbi.nlm.nih.gov/snp/rs371156848). This variant is seen eight times only in a heterozygous state (https://gnomad.broadinstitute.org/variant/11‐134131054‐C‐A?dataset=gnomad_r2_1). Interestingly this mutation has not yet been reported in patients, therefore the phenotypical effects are still unknown. Since the available evidence is currently insufficient to determine the role of this variant in disease, it has been classified as a Variant of Uncertain Significance in ClinVar (https://www.ncbi.nlm.nih.gov/clinvar/variation/1001998/). In the protein, the Asn274 residue is highly conserved across species, and a moderate physicochemical difference occurs in Asn to Lys substitution. The Asn274 residue is positioned in alfa helix G,^32^ which involves the substrate binding region (274–277 according to the Nextprot database, based on the study by Battaile et al.^32^) (Figure S2d), therefore, we expect it to be relevant for the enzymatic function of the homotetramer. Consistently, this variant is predicted to be probably damaging and deleterious *in silico* by both PolyPhen‐2 and SIFT. Similar predictions derive from advanced modeling of protein sequence and biophysical properties reported by Invitae (Accession: SCV001487418.1, submitted: [January 7, 2021]).

### 3.3 Implications on flavin homeostasis

Despite mutational analysis of Rf homeostasis connected genes (*SLC52A1*/*SLC52A2*/*SLC52A3*, *FLAD1*, or *SLC25A32*) did not reveal any pathogenic variants, we investigated the Rf status in the patient and controls, by evaluating both plasma vitamin/vitamin‐derivative concentrations by HPLC, and FAD abundance through erythrocyte glutathione reductase (EGR) enzymatic assays.

Table 2 shows plasmatic acid‐extractable flavin profiles obtained by HPLC at a different stage of therapy of the patient; these values are compared with age‐matched controls. As expected, FAD is the main species detectable (85% of the total content) in the plasma extracts, due to the activity of blood FAD synthase^33^, ^34^, ^35^, ^36^; on the other hand, only small amounts of Rf and FMN are found. In the patient, at the first decompensation (P ^1st^D_adm_), a significant decrease of both FAD and FMN levels is found compared to controls; a slight, even if not significant, decrease in Rf level is also observed. At the second decompensation (P ^2nd^D_adm_), following treatment with CoQ10 and l‐carnitine, FAD values appear to come back to normality. FMN and Rf increased, but they still do not reach the normality. At the start of Rf treatment during the second hospitalization, Rf, but not FMN level, increases, entering the normal interval. Following continuous treatment with Rf, an excess of the vitamin can be observed in the plasma (eight times more than at the first admission). FAD content remains stable and in the normal range, as well as FMN value appeared to be compensated.

**TABLE 2:** Plasma flavin concentration (nmol/L)

		FAD	FMN	Rf	Tot.
Controls (*n* = 8)	Interval	35.0–49.6	7.4–14.5	3.8–22.4	46.7–88.3
	Mean ± SEM	44.0 ± 2.1	9.3 ± 0.9	7.7 ± 2.1	61.0 ± 4.6
P ^1st^D_adm_		37.8 ± 1.4*	3.5 ± 0.1***	2.9 ± 0.1	44.2 ± 1.5**
P ^2nd^D_adm_		49.7 ± 0.5	5.1 ± 0.3**	3.1 ± 0.0	57.9 ± 0.2
P _at start of Rf treatment_		49.6 ± 3.3	4.5 ± 0.4***	6.5 ± 0.1	60.6 ± 3.6
P _2 months after start of Rf treatment_		43.3 ± 1.2	8.1 ± 0.4	24.7 ± 0.4	76.0 ± 1.3

*Note*: Data represent the mean ± SEM of two different HPLC determinations; Student's *t*‐test: *p < 0.05; **p < 0.01.

The spectrophotometric enzymatic assay, introduced as EGRAC, is based on the ability of externally added FAD to reactivate the apo‐enzyme, present in erythrocyte ghosts in case of ipo‐vitaminosis (deficiency status = ≥1.4, marginal deficiency status = 1.2–1.4, acceptable status = ≤1.2). Different from analytic methods, this approach refers to a medium‐term analysis of the nutritional status of Rf,^27^, ^37^ or anyway FAD availability.

As reported in Table 3, in patient EGRAC was 3.2 ± 0.2 at the first decompensation and 2.6 ± 0.2 at the second metabolic decompensation, suggesting a severe Rf/FAD scarcity. At the start of Rf treatment during the second hospitalization, the EGRAC value significantly decreased. Then, following continuous Rf therapy, the EGRAC value (1.2 ± 0.1) indicated an acceptable FAD status. Endogenous enzymatic rates of EGR (measured in the absence of externally added FAD during the assay) are also reported in Table 3.

**TABLE 3:** EGRAC and endogenous EGR activity

	EGRAC^a^	Endogenous EGR rate (μmol/min·g Hb)
P ^1st^D_adm_	3.2 ± 0.2	1.0 ± 0.1
P ^2nd^D_adm_	2.6 ± 0.2	2.8 ± 0.1
P _at start of Rf treatment_	1.4 ± 0.0	4.6 ± 0.1
P _2 months after the start of Rf treatment_	1.2 ± 0.1	5.1 ± 0.2

*Note*: Data represent the mean ± SEM of three different determinations.

Abbreviations: EGR, erythrocyte glutathione reductase; EGRAC, erythrocytes glutathione reductase activation coefficient.

^a^ Deficiency status ≥ 1.4, marginal deficiency status = 1.2–1.4, acceptable status ≤ 1.2.

Both analytical and enzymatic results demonstrate a profound alteration of Rf homeostasis in this patient at the first decompensation and a reversal of this biochemical phenotype in response to first treatments and, even better, to high and continuous supplementation with Rf. As far as genetical analysis revealed, Rf homeostasis defects are not congenital, but probably due to a dietary deficiency. Nevertheless, we cannot exclude a secondary defect that may be related to oxidative metabolic stress.^38^

### 3.4 RFVT2level in patient erythrocytes

To further substantiate Rf homeostasis impairment, the effort was done, as far as we know for the first time, to measure the levels of Rf transporters (RFVT1–3) in membrane‐enriched fractions from erythrocytes by Western blotting. The protein amounts detectable in this way are expected to be the result between the rate of protein expression in erythroblasts and the rate of protein turnover during erythrocyte life.

**FIGURE 2:**
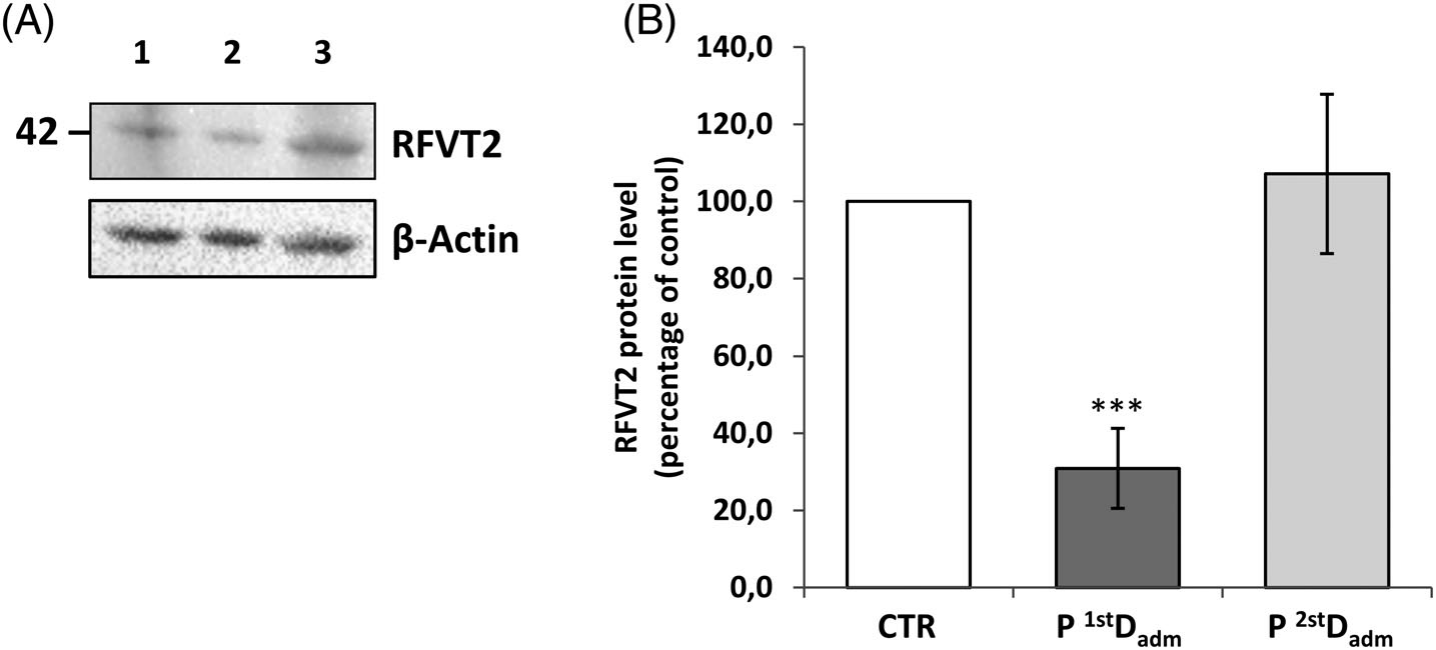
Representative immunoblot analysis of RFVT2 protein. (A) Protein extracts from nonsoluble fractions of erythrocytes from one age‐matched CTR (Lane 1), patient at the first (P ^1st^Dadm, Lane 2) and at the second decompensation (P ^2nd^Dadm, Lane 3) were loaded on sodium dodecyl sulfate–polyacrylamide gel electrophoresis (SDS‐PAGE) and immunoblotted with a polyclonal antibody raised against RFVT2, and a monoclonal antibody against β‐actin, used as loading control. (B) Relative amount of RFVT2 in patient with respect to control was quantified and normalized to β‐actin. The error bars represent standard error of the mean (SEM) of four different determinations (Student *t*‐test: *p < 0.05, ***p < 0.001)

A screening made of the product of the three genes *SLC52A1–3*, revealed the sole presence of RFVT2 (Figure 2). The RFVT2 cross‐reactive band migrates at about 42 kDa in SDS‐PAGE, in good agreement with the theoretical molecular mass of RFVT2 (45.7 kDa, according to the EXPASY PROTPARAM tool). In the same extracts, no cross‐reactive bands for RFVT1 and RFVT3 at the expected molecular weight (46.3 and 50.8 kDa, respectively) were detectable. A significant decrease of the RFVT2 band was found in patient membranes at the first decompensation, but not at the second one, maybe because of the beneficial effect of the therapy.

## 4 DISCUSSION

We introduced here a novel Rf‐responsive patient, additionally and successfully treated also with the ketone body 3OH‐butyrate. He was born before neonatal screening for both MADD and IBD deficiency (IBDD; OMIM #611283) were introduced in Italy by Law (Disposizioni in materia di accertamenti diagnostici neonatali obbligatori per la prevenzione e la cura delle malattie metaboliche ereditarie. GU n.203 del 31‐08‐2016), therefore the diagnosis was possible only after clinical manifestations.

In this boy during the metabolic crisis, organic acids and acylcarnitine profile resembling late‐onset MADD led us to suspect mutations in one of the genes currently associated with this pathology, that are linked either to defects in FAD reoxidation (ETF/ETF:QO system) or defects in FAD synthesis/transport. Genetic analysis, indeed, revealed the presence of a single mutation in *ETFDH* exon 5, which, however, is expected not to be responsible *per se* for the biochemical altered phenotype.^29^ Thus, in the absence of a further sequencing or deep intronic regions,^39^, ^40^ the first hypothesis of late‐onset MADD, based on the clinical and biochemical profile, can be neither ruled out nor confirmed. Nevertheless, we searched for other factors, or a digenic constellation combined with *ETFDH* heterozygosity to possibly explain this complex phenotype. Quite interestingly, we found compound heterozygous mutations (being the variant c.822C>A never described before) in one member of the ACAD family, that is, the *ACAD8* gene, which is potentially responsible for IBDD.

*ACAD8* gene codes for the tetrameric mitochondrial located enzyme, IBD, principally involved in valine metabolism. Like other ACADs, it is expected to directly interact with the “funnel” of electrons, that is, the ETF/ETF:QO system on the internal face of the inner mitochondrial membrane (Figure S1).^5^, ^6^ Even though the contribution of *ACAD8* compound heterozygosity to the patient's MADD resembling phenotype is difficult to establish, we can speculate that the occurrence of an expected impaired ETF:QO activity, concomitant with a predictable decreased functionality of IBD, physically interacting through ETF in the membrane (Figure S1), possibly exacerbated the biochemical phenotype in this boy.

Confirmations by other patients, a more detailed analysis of intronic regions, or hopefully the programmed development of a suitable cell model, will confirm our hypothesis that IBD impairment combined with *ETFDH* heterozygosity could be sufficient to generate MADD phenotype.

With the discussed limitation to consider him a case of IBDD, as far as we know, this is the first symptomatic patient described in the South of Italy, being the other one, described in the study by Scolamiero et al.,^41^ asymptomatic. The latter patient was described by the application of targeted metabolomics in the preclinical diagnosis of inborn errors of metabolism in the Campania region of southern Italy between 2007 and 2014, with an estimated IBDD prevalence of 1/45 466.^41^

The first patient with IBDD was described by Roe et al.^18^; since the age of 12 months, he presented dilated cardiomyopathy, anemia, and carnitine deficiency. In the plasma acylcarnitine profile, an elevated C4‐acylcarnitine was found. Urine isobutyrylglycine (IBG) levels (subsequently considered additional supporting evidence of IBDD^42^) were not measured in this patient. Treatment with l‐carnitine was beneficial. Since then, patients with inherited IBDD have mostly been diagnosed through newborn screening programs due to elevated C4‐acylcarnitine levels and have appeared clinically asymptomatic, though a few patients have shown speech delay and neurodevelopmental delay/intellectual disability, hypotonia, emesis, failure to thrive (see Sass and colleagues^42^, ^43^, ^44^, ^45^, ^46^ and references therein). Only a few patients, who were not diagnosed from a newborn screening program, as our patient was, had anemia, dilated cardiomyopathy, ketotic hypoglycemia, and asthma.^18^, ^43^, ^47^

In agreement with *ACAD8* deficiency, in acylcarnitine profiles of our patient the levels of C4 and C4 related ratios are significantly altered, reaching values more pronounced than in a large cohort of IBDD subjects, recently described,^44^ and even higher than the levels of the other MADD specific acylcarnitines (see Mereis et al.^16^). If actually due to a reduction in IBD activity, this complex biochemical phenotype might be explained by the speculation, which is still a matter of debate, that IBD might overlap SCAD functionality.^48^, ^49^, ^50^ Notably, in our patient IBG was not detected in the urine. Indeed, its increase was not always reported, as in the case of severalrecently described patients.^43^, ^44^

Interestingly ETF/ETF:QO, IBD, and other ACADs requires FAD for their enzymatic activities; it is noteworthy that we revealed in our patient a disturbance of flavin cofactor availability, presumably due to fasting, as revealed by EGRAC and HPLC measurements. FAD scarcity is expected to induce defects in downstream enzymes. Thus, another supporting evidence of FAD scarcity in this patient is the hyperprolinemia we detected at the time of first decompensation, consistent with a mitochondrial flavoenzyme proline dehydrogenase deficiency.^51^ Indeed, an alimentary maternal Rf deficiency has been described in other infants as responsible for transient and moderate VLCAD deficiency.^52^

Moreover, FAD scarcity can induce ETF/ETF:QO system instability,^53^ and this condition might generate ROS, key regulators of several cellular responses.^38^ Thus, the scarcity of flavin cofactor may exacerbate the patient's MADD phenotype or, even, trigger severe initial metabolic decompensation. In this frame, the slight hyperasparaginemia we detected might result from a transcriptional response involving asparagine synthetase.^54^

Altered RFVT2 levels we detected in erythrocytes (Figure 2) might be the consequences of these cellular stress conditions. Indeed, the proposal that mutations of ETF:QO in MADD might, in turn, induce alteration of FAD homeostasis has been launched by others^55^, ^56^; further research is necessary to establish this relationship at the molecular level, possibly using *Caenorhabditis elegans* models^57^ or better cell models from our patient.

Whatever the mechanism is, derangements in Rf transport are expected to result in an increased cellular Rf demand and they constitute a starting point also to address the molecular rationale for Rf therapy in our patients.

Fortunately, patient symptoms were successfully treatable with continuous Rf (l‐carnitine and CoQ10) supplementation. In the attempt to bypass liver beta‐oxidation defects and to ameliorate energetic supply to extra‐hepatic tissue, a nutritional therapy based on low intake of long‐chain lipids and 3OH‐butyrate supplementation was recently introduced, as suggested by Mereis et al.,^16^ and the amelioration of biochemical pattern confirms the impairment in the beta‐oxidation pathway.

This case demonstrates that, in the event of biochemical derangements suggestive of Rf metabolism impairment, even in the absence of a direct genetic defect, further analysis on Rf homeostasis may reveal secondary and possibly treatable alterations.

## CONFLICTS OF INTEREST

The authors declare no conflicts of interest.

## AUTHOR CONTRIBUTIONS

**Albina Tummolo:**Responsible for clinical management of the patient, contributed to draft the paper and revise it critically. **Piero Leone:** Planned and conducted Western blotting experiments, contributed to draft the paper. **Maria Tolomeo:** Planned and conducted HPLC experiments, contributed to critical revision of the paper. **Rita Solito:** Conducted and analyzed enzymatic assays. **Matteo Mattiuzzo:** Performed genetic analyses. **Francesca Romana Lepri:** Responsible for genetic analyses, contributed to critical revision of the paper. **Tania Lorè** and **Roberta Cardinali:** Conducted and analyzed biochemical plasma and urine assays. **Donatella De Giovanni:** Responsible for clinical management of patient. **Simonetta Simonetti:** Responsible for conception and interpretation of biochemical analyses. **Maria Barile:** Participated to conception of the experimental plan, to draft the paper, and to provide funding acquisition. All authors have read and agreed to the published version of the manuscript.

## ETHICS STATEMENT

All procedures followed were in accordance with the ethical standards of the responsible committee on human experimentation (institutional and national) and with the Helsinki Declaration of 1975, as revised in 2000. Informed consent was obtained from the patient for being included in the study.

## Supporting information


**Appendix S1** Supporting Information

**FIGURE S1 Schematic representation of mitochondrial valine metabolism.** On the right, mitochondrial valine oxidation pathway and the enzymes involved. On the left, a magnification of the proposed physical interaction between IBD and ETF/ETF:QO system. Names of enzymes are indicated in the figure as listed below: transaminase (EC 2.6.1._); BCKAD: branched‐chain‐2‐oxoacid decarboxylase (EC 4.1.1.72); IBD (ACAD8): isobutyryl‐CoA dehydrogenase (EC 1.3.8._); ETF:QO: electron transfer flavoprotein ubiquinone oxidoreductase (EC 1.5.5.1); ETF: electron transfer flavoprotein; crotonase (EC 4.2.1.17); HIBCH: 3‐hydroxyisobutyryl‐CoA hydrolase (EC 3.1.2.4); HIBADH: 3‐hydroxyisobutyrate dehydrogenase (EC 1.1.1.31); MMSDH: methylmalonate‐semialdehyde dehydrogenase (EC 1.2.1.27).

**FIGURE S2 Localization of mutations in *ACAD8* gene and in IBD protein.** (a) Schematic representation of *ACAD8* gene structure with exons (boxes E1‐E11) and introns (lines between boxes) showing the localization of the investigated gene variations (c.512C > G; c.822C > A). (b) Structure of isobutyryl‐CoA dehydrogenase: ribbon representation of isobutyryl‐CoA dehydrogenase protein based on the crystal structure of human IBD (PDB ID: 1RX0).^32^ In the insets, zoom into the IBD chain A, showing Ser171Cys (c) and Asn274Lys (d) natural mutations.

## Data Availability

Data supporting genetic analysis were derived from the following resources which are available in public domains: dbSNP database (https://www.ncbi.nlm.nih.gov/snp/); Clin Var (https://www.ncbi.nlm.nih.gov/clinvar/); Genome Aggregation Database (https://gnomad.broadinstitute.org); Combined Annotation Dependent Depletion (https://cadd.gs.washington.edu/) V.1.3; Sorting Intolerant from Tolerant (SIFT) (https://sift.bii.a-star.edu.sg/); PolymorphismPhenotyping v2 (PolyPhen‐2) (http://genetics.bwh.harvard.edu/pph2/); Nextprot database (https://www.nextprot.org/).
