# Supplementary figures and images for: Combined isobutyryl‐CoA and multiple acyl‐CoA dehydrogenase deficiency in a boy with altered riboflavin homeostasis
Source: JIMD Rep. 2022 May 7;63(4):276–91. doi: 10.1002/jmd2.12292 (PMC9259400; doi:10.1002/jmd2.12292)

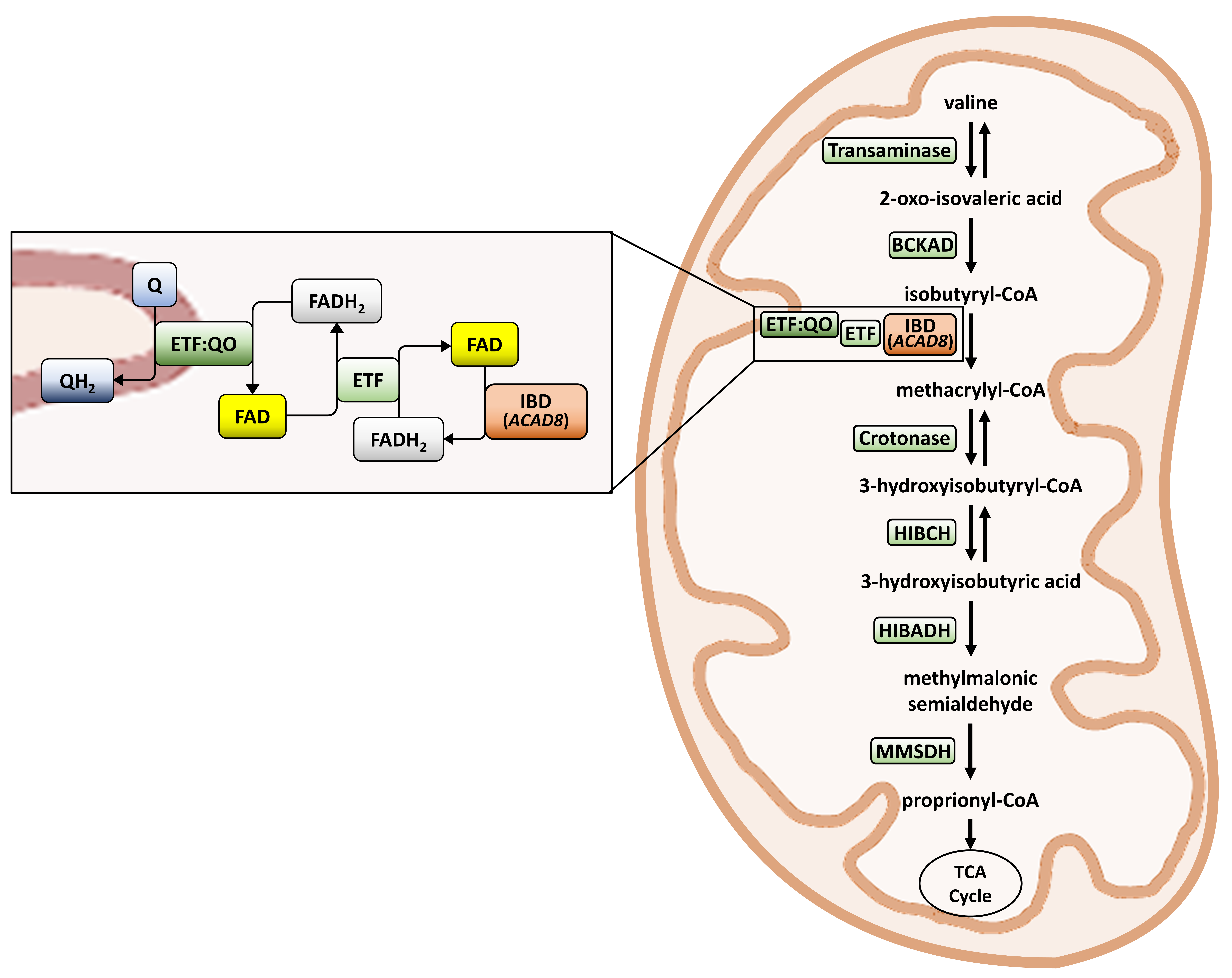

Supplement: Supplementary file 2 — FIGURE S1 Schematic representation of mitochondrial valine metabolism. On the right, mitochondrial valine oxidation pathway and the enzymes involved. On the left, a magnification of the proposed physical interaction between IBD and ETF/ETF:QO system. Names of enzymes are indicated in the figure as listed below: transaminase (EC 2.6.1._); BCKAD: branched‐chain‐2‐oxoacid decarboxylase (EC 4.1.1.72); IBD (ACAD8): isobutyryl‐CoA dehydrogenase (EC 1.3.8._); ETF:QO: electron transfer flavoprotein ubiquinone oxidoreductase (EC 1.5.5.1); ETF: electron transfer flavoprotein; crotonase (EC 4.2.1.17); HIBCH: 3‐hydroxyisobutyryl‐CoA hydrolase (EC 3.1.2.4); HIBADH: 3‐hydroxyisobutyrate dehydrogenase (EC 1.1.1.31); MMSDH: methylmalonate‐semialdehyde dehydrogenase (EC 1.2.1.27). [file JMD2-63-276-s002.tif]

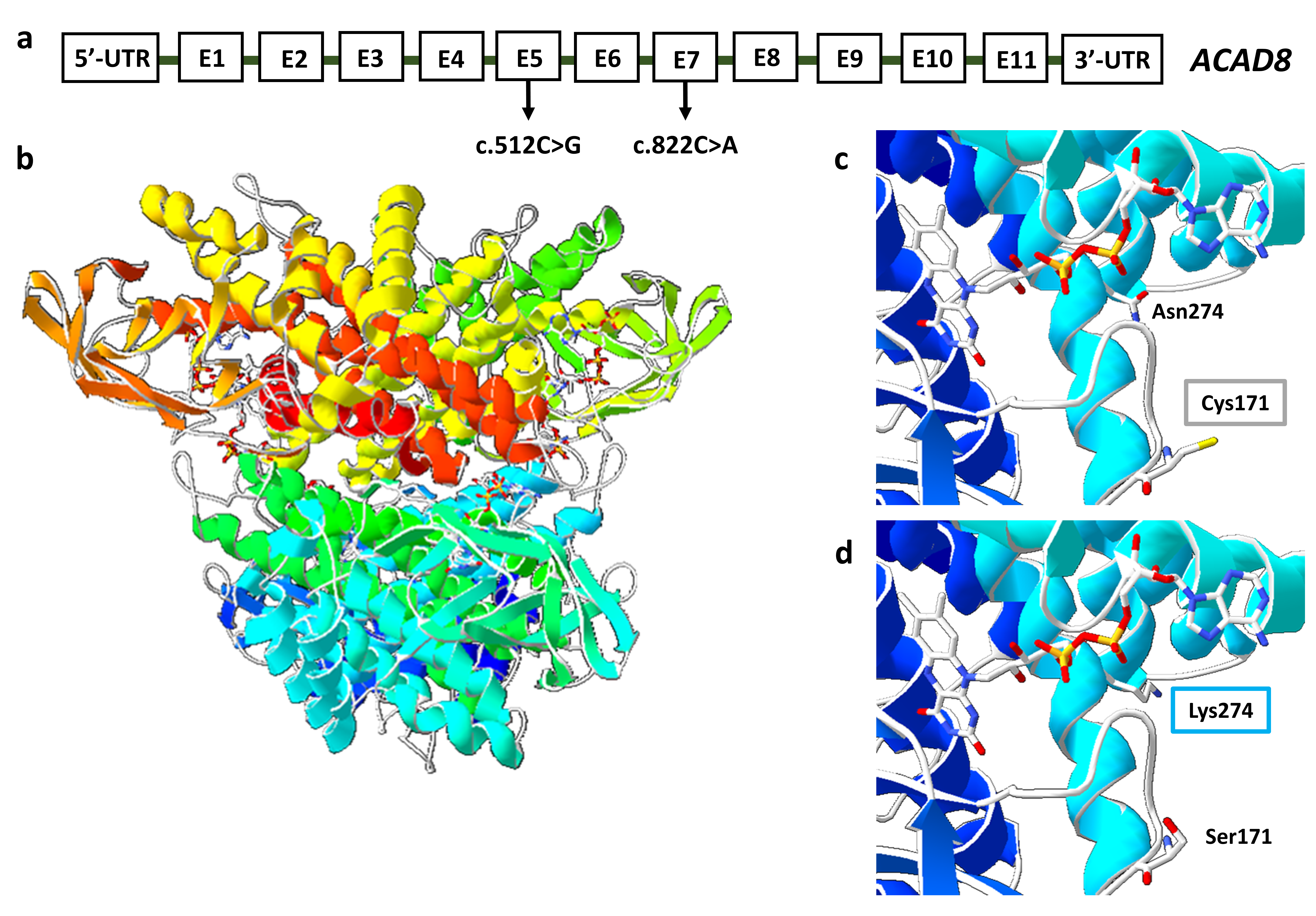

Supplement: Supplementary file 3 — FIGURE S2 Localization of mutations in ACAD8 gene and in IBD protein. (a) Schematic representation of ACAD8 gene structure with exons (boxes E1‐E11) and introns (lines between boxes) showing the localization of the investigated gene variations (c.512C > G; c.822C > A). (b) Structure of isobutyryl‐CoA dehydrogenase: ribbon representation of isobutyryl‐CoA dehydrogenase protein based on the crystal structure of human IBD (PDB ID: 1RX0).32 In the insets, zoom into the IBD chain A, showing Ser171Cys (c) and Asn274Lys (d) natural mutations. [file JMD2-63-276-s003.tif]
